# Th17 Activation and Th17/Treg Imbalance in Prolonged Anterior Intraocular Inflammation after Ocular Alkali Burn

**DOI:** 10.3390/ijms23137075

**Published:** 2022-06-25

**Authors:** Miner Yuan, Xiaobing Qian, Yanqiao Huang, Xinqi Ma, Fang Duan, Yao Yang, Bingsheng Lou, Xiaofeng Lin

**Affiliations:** State Key Laboratory of Ophthalmology, Zhongshan Ophthalmic Center, Sun Yat-sen University, 54 Xianlie Road, Guangzhou 510060, China; freeflying@126.com (M.Y.); qianxiaobing2008@163.com (X.Q.); huangyanqiao@gzzoc.com (Y.H.); xqma0708@163.com (X.M.); duanfangg@126.com (F.D.); yangyao22@163.com (Y.Y.); 13450475056@163.com (B.L.)

**Keywords:** Th17 activation, Th17/Treg imbalance, ocular alkali burn, anterior intraocular inflammation

## Abstract

Ocular alkali burn (OAB) is a sight-threatening disease with refractory ocular inflammation causing various blinding complications. Th17 lymphocytes account for the pathogeneses of the autoimmune disease and chronic inflammation, but their role in prolonged anterior intraocular inflammation after OAB is still unknown. A rat OAB model was established for this purpose. Anterior intraocular inflammation was observed in both the acute and late phases of OAB, and histological examination confirmed the presence of inflammatory cell infiltration and fibrin exudation in the anterior segment. Luminex xMAP technology and qPCR were used to evaluate the intraocular levels of cytokines. The levels of IL-1β, IL-6, and TNF-α were significantly elevated during the acute phase. The expression of IL-17A gradually increased from day 7 onwards and remained at a relatively high level. Immunofluorescence was performed to identify Th17 cells. CD4 and IL-17A double positive cells were detected in the anterior chamber from days 7 to 28. Flow cytometry showed that the frequency of Th17 cells increased in both lymph nodes and spleen, while the frequency of Treg cells remained unchanged, resulting in an elevated Th17/Treg ratio. The present study suggests that Th17 activation and Th17/Treg imbalance account for prolonged anterior intraocular inflammation after OAB.

## 1. Introduction

Ocular alkali burn (OAB) is a common blinding disease in the working-age population, causing extensive damage to the ocular surface and anterior segment of the eye. Visual prognosis in severe cases of OAB is usually poor [1,2,3,4], because of numerous complications, including corneal opacity and perforation, corneal neovascularization, symblepharosis, glaucoma, cataract, and retinal ganglion cell damage [5,6,7,8]. It was believed decades ago that injuries to the ocular surface and intraocular tissue result from the direct impact of alkaline stimuli [9]. Permanent damage to the cornea, iris, or lens can occur when the pH of the aqueous humor exceeds 11. However, Paschalis et al. showed that the pH value in the anterior chamber exceeded 11 within 1 min after 1N sodium hydroxide exposure and returned to normal within 45 min in mice and 180 min in rabbits [6]. They also determined that the pH in the vitreous cavity never did not increase within 26 h in either mice or rabbits [6]. Hence, rather than the alkali itself, some other pathologic factors, such as inflammation, could account for the continuous irritation to the anterior segments in the later phase of OAB.

The clinical progress of OAB is divided into the following four phases: immediate (day 0), acute (days 0–7), early reparative (days 7–21), and late reparative (after day 21) [10]. Acute inflammation plays a critical role within 7 days, contributing to ocular surface melting, with the prompt elevation of the pro-inflammatory cytokines IL-1β, IL-6 and TNF-α [11]. Prolonged inflammation and scarring occur in the early reparative stage and last until the late phase, resulting in multiple morphological and functional disorders of the cornea, iris, trabecular meshwork, lens, ciliary body, retina, and optic nerve [5,6,7,11,12,13,14,15,16]. In histopathological studies, T and B lymphocytes were observed after the acute infiltration of neutrophils and monocytes in rabbit alkali-burned corneas and lasted over 2 weeks. This indicates underlying chronic inflammation and the participation of adaptive immunity during the reparative stages [16]. Denatured corneal proteins may act as antigens during this process [16,17]. In a rat OAB model, macrophages and dendritic cells expressing MHC-II molecules, serving as antigen-presenting cells (APC), were found in the iris 2–3 days after modeling and were observed until the late reparative stage [18]. As a result, CD4^+^ T lymphocytes gathered in the anterior segment beginning on day 7 and persisted throughout the reparative phase (day 14–30) [18]. Persistence of APCs and accumulation of CD4^+^ T lymphocytes increased the probability of excessive intraocular immunity and autoimmune responses.

Over the past two decades, the Th17 lymphocyte population has been confirmed to play pro-inflammatory roles in cell-mediated autoimmune diseases such as psoriasis, rheumatoid arthritis, and ankylosing spondylitis [19,20,21]. In contrast, Tregs act as anti-inflammatory regulators and inhibit autoimmune responses by preventing T and B lymphocyte activation and proliferation [22,23,24,25]. Th17 cells cause or exacerbate chronic and autoimmune inflammation by secreting their major effector IL-17. Treg cells suppress autoimmunity by producing anti-inflammatory cytokine IL-10. Th17 and Treg lymphocytes share a common precursor cell (naïve CD4 T cells) and a common signaling pathway mediated by TGF-β in differentiation and regulation [26]. Thus, Th17/Treg balance is crucial for maintaining immune homeostasis. Any disturbance in this balance would result in the disruption of immune tolerance and induction of an excessive immune response. Therefore, the Th17/Treg ratio, reflecting Th17/Treg balance, can predict the fate of some autoimmune diseases and chronic inflammation [27,28,29].

In ophthalmology, studies on Th17 lymphocytes were mainly focused on the pathogenesis of autoimmune uveitis such as Vogt-Koyanagi-Harada disease (VKH), sympathetic ophthalmia, Bechet’s disease, sarcoidosis, and “birdshot” chorioretinopathy as well as the progression of Sjogren’s syndrome, late-term corneal allorejection, and the recurrence of herpetic keratitis [30,31,32,33,34]. The relative insufficiency of the Treg cell population, either in frequency or function, could result in the activation of VKH and Sjogren’s syndrome [32,35]. Th17/Treg imbalance has been reported to be responsible for the pathogenesis and aggravation of experimental autoimmune uveitis (EAU) [36]. Reversion of the Th17/Treg ratio can alleviate inflammatory injury and promote recovery in the EAU model [37,38]. As mentioned above, APCs and CD4^+^ T lymphocytes were identified in the anterior segment of OAB rats in early and late reparative stages, indicating the existence of prolonged and excessive intraocular inflammation [18]. It is still unknown whether Th17 cells are involved in prolonged anterior intraocular inflammation after OAB or how the Th17/Treg ratio changes during prolonged anterior intraocular inflammation after OAB. In this study, we aimed to determine whether Th17 lymphocytes were involved in prolonged anterior intraocular inflammation after OAB and whether the Th17/Treg balance was disturbed in prolonged anterior intraocular inflammation induced by ocular alkali burn.

## 2. Results

### 2.1. Prolonged Anterior Intraocular Inflammation Observed in Rat OAB Model

**Figure 1:**
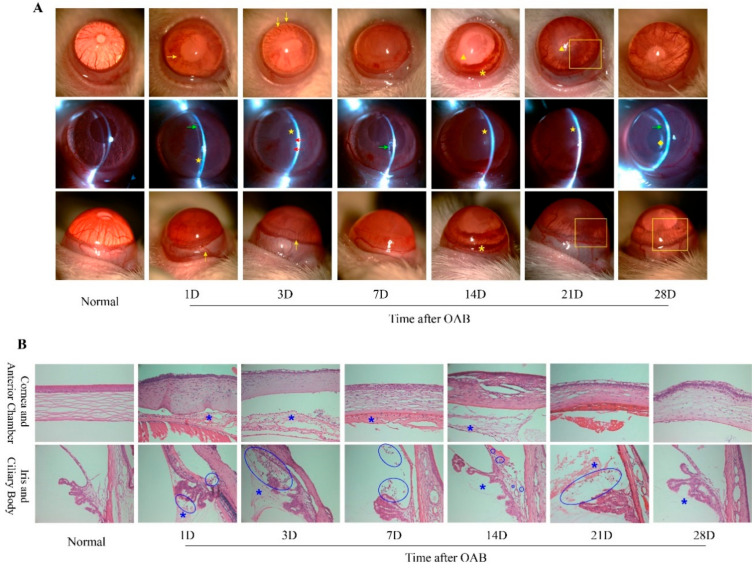
Slit lamp photographs and H and E staining of the anterior segment of the normal control and OAB-subjected eyes (*n* = 6 for each time point). (**A**): Slit lamp photographs of the anterior segment of the normal control and Ocular Alkali Burn (OAB-subjected eyes on the 1st, 3rd, 7th, 14th, 21st, and 28th day post-modeling. Ocular hyperemia and dilated vessels (yellow →) in the conjunctiva, limbus, and iris, aqueous flare (yellow ★), anterior cells (red →), fibrin exudation (green →), posterior synechia and irregular pupil (yellow ▲), anterior chamber hyphema (yellow *), corneal neovascularization (yellow □), fibrin membrane, and complicated cataracts (yellow ◆) were observed at different timepoints. (**B**): Histopathological slices with H&E staining of the anterior segment of normal control and OAB-subjected eyes at each timepoint. Inflammatory cells were not detected in the cornea, iris, anterior, or posterior chambers in the normal control eye(s). Various immune cells (blue ○) and fibrin exudation (blue *) were observed in the anterior segment from days 1 to 21. On the 28th day, immune cells were not found, but fibrin exudation remained.

The anterior intraocular inflammation induced by OAB was observed at each timepoint after modeling, including ocular hyperemia, aqueous flare (AF), anterior chamber (AC) cells, fibrin exudation, posterior synechia, and irregular pupils. In the acute stage, AF and AC cells were remarkable in most eyes, followed by fibrin exudation around the pupils. On the 7th day, acute inflammation gradually faded, and posterior synechia or the occlusion of pupil could be seen in some experimental eyes. Neovascularization, recurrent aqueous humor opacity, and mild anterior bleeding in some individuals were observed during the second and third weeks after OAB. At the end of the fourth week, anterior inflammation decreased again, and fibrin membranes, irregular pupils, or complicated cataracts were observed in some OAB-subjected eyes. The findings in the later phase of OAB indicate that prolonged anterior intraocular inflammation did exist and might have lasted for several weeks. Representative slit-lamp photographs of the control and subject eyes at each time point are shown in Figure 1A. H and E staining showed that inflammatory cell infiltration and fibrin exudation occurred in both the anterior and posterior chambers, and sometimes in the stroma of the iris and the ciliary body, from day 1 to day 21 after OAB (Figure 1B). This pathological manifestation also supports the notion that prolonged anterior intraocular inflammation occurred after OAB.

### 2.2. Elevated Intraocular IL-17A Level after the Acute Phase of OAB

**Figure 2:**
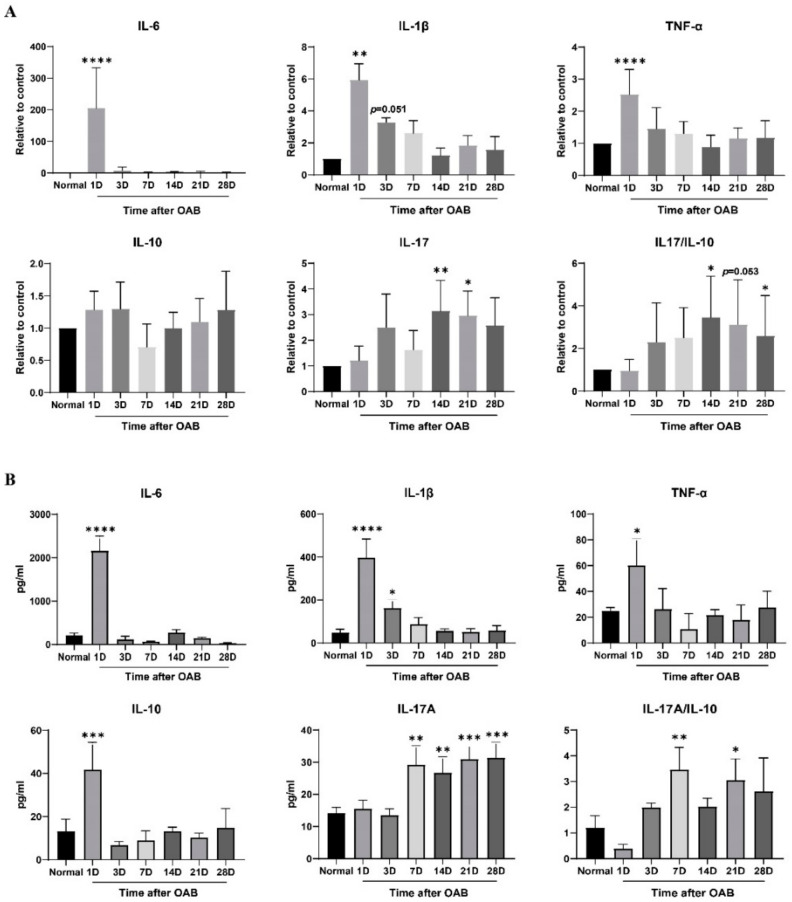
Intraocular levels of IL-1β, IL-6, TNF-α, IL-10, and IL-17A, and the IL-17A/IL-10 ratio of normal control and OAB subjected eyes. (**A**): mRNA levels of IL-1β, IL-6, TNF-α, IL-10, and IL-17A, and the IL-17A/IL-10 ratio in the ciliary body of normal control and OAB-subjected eyes on the 1st, 3rd, 7th, 14th, 21st, and 28th day after alkaline exposure. (**B**): Protein expression levels of IL-1β, IL-6, TNF-α, IL-10, and IL-17A, and the IL-17A/IL-10 ratio in the intraocular fluid of normal control and OAB-subjected eyes at each timepoint. Data are presented as mean ± SD (*n* = 6, * *p* < 0.05, ** *p* < 0.01, *** *p* < 0.001, **** *p* < 0.0001 versus the control group).

To identify the factors responsible for prolonged anterior intraocular inflammation after OAB, the transcription and protein levels of IL-1β, IL-6, TNF-α, IL-10, and IL-17A were measured (Figure 2). The transcription of IL-1β, IL-6, and TNF-α increased dramatically in the first 3 days, with a peak on the first day after OAB modeling (*p* < 0.05), and then returned to near-normal from the 7th day to the 28th day (*p* > 0.05). The transcription level of IL-10 remained unchanged (*p* > 0.05), while that of IL-17A increased from day 14 to day 21 (*p* < 0.05). The expression levels of IL-1β, IL-6, and TNF-α showed nearly the same trend as their respective transcription levels, increasing within the first 3 days and returning to normal from the 7th to the 28th day. The expression level of IL-10 increased significantly on the first day (*p* < 0.05) and declined rapidly soon afterwards. The expression of IL-17A increased from day 7 onwards and remained at a relatively high level until the end of the observation period (*p* < 0.05). Thus, the increased IL-17A/IL-10 ratio in the later phase of OAB was mainly related to the elevation of IL-17A, rather than the reduction of IL-10.

### 2.3. Th17 Lymphocytes Detected in the Anterior Segment of OAB-Subjected Eyes

**Figure 3:**
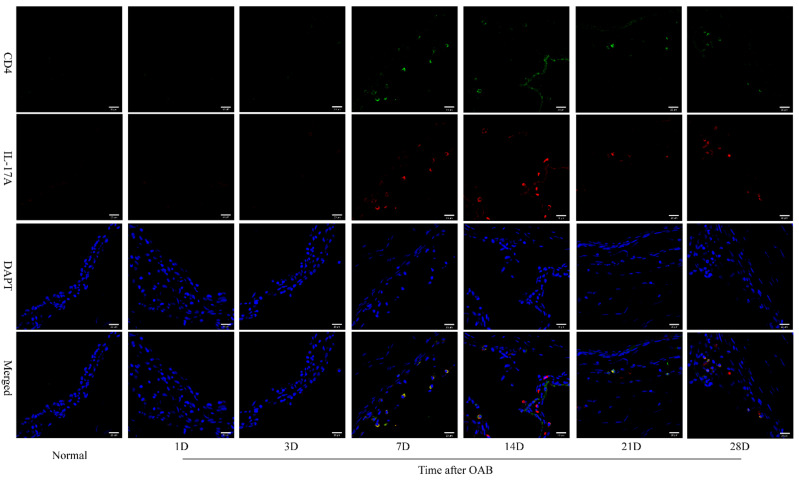
Immunofluorescence examination of normal control and OAB-subjected eyes (*n* = 6 for each time point). Immunofluorescent staining of CD4 (green) and IL-17A (red) in the normal control and OAB-subjected eyes on the 1st, 3rd, 7th, 14th, 21st, and 28th day post modeling (40X). Cell nuclei were stained with DAPI (blue). CD4 and IL-17A double positive cells were found around the iris from days 7 to 28. Scale bar: 20 μm.

Immunofluorescence staining was performed to determine whether Th17 lymphocytes, the main source of IL-17A, were involved in the process of prolonged anterior intraocular inflammation after OAB. CD4 and IL-17A double positive cells, which were thought to be Th17 lymphocytes, could be detected mainly around the surface of the iris in OAB-subjected eyes from day 7 to day 28 (Figure 3). It was found that Th17 lymphocytes might partly account for anterior intraocular inflammation in the later phase of OAB. Negative control photos are shown in Supplementary Figure S1.

### 2.4. Increased Frequency of Th17 Population and Elevated Th17/Treg Ratio Rat OAB Model

**Figure 4:**
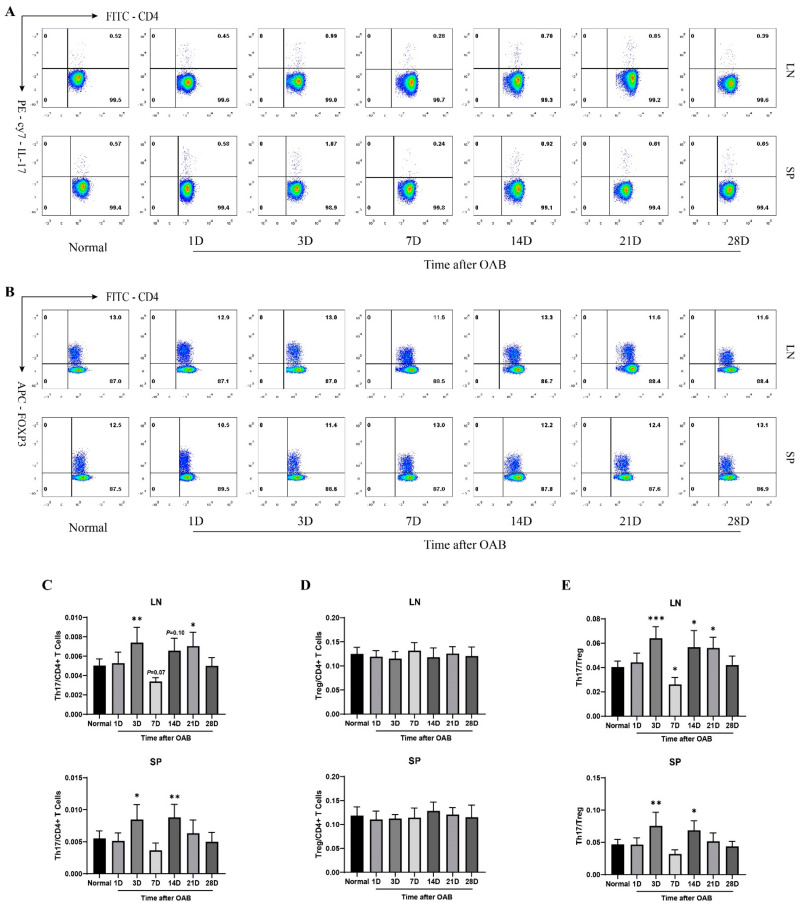
Dynamic changes in the frequencies of Th17 and Treg populations, and Th17/Treg ratio in lymph nodes and spleen after OAB. A and B: Flow cytometry analysis of Th17 (**A**) and Treg (**B**) cells in submaxillary and cervical lymph nodes (LN) and spleen (SP) of normal control and OAB-subjected eyes on the 1st, 3rd, 7th, 14th, 21st, and 28th day post-modeling. C, D, and E: Statistical analysis of the frequencies of Th17 (**C**), Treg (**D**), and Th17/Treg ratio (**E**) in LN and SP. Data are presented as mean ± SD (*n* = 6, * *p* < 0.05, ** *p* < 0.01, *** *p* < 0.001 versus the control group).

To investigate the dynamic changes in the levels of Th17 and Treg cells during OAB, flow cytometry was performed at each time point before and after OAB modeling (Figure 4). The frequency of Th17 cells increased significantly on days 3 and 21 (*p* < 0.05). The level of Treg cells remained nearly unchanged in the lymph nodes (*p* > 0.05). The variation in Th17 and Treg levels showed similar trends in spleen, and the frequency of Th17 cells increased on days 3 and 14 (*p* < 0.05). The Treg cells level did not change at any timepoint after OAB compared to the normal control (*p* > 0.05). Accordingly, the Th17/Treg ratio increased early on the 3rd day after OAB in both lymph nodes and spleen. The ratio then decreased on the 7th day, increased again on the 14th day in lymph nodes and spleen, and remained high in lymph nodes on the 21st day (*p* < 0.05).

## 3. Discussion

In the present study, we first confirmed the existence of prolonged anterior intraocular inflammation in the early and late reparative stages in a rat ocular alkali burn (OAB) model. We then demonstrated that the elevated expression of intraocular IL-17A, the main pro-inflammatory effector of Th17 cells, remained at a relatively high level during the reparative stages. We detected the infiltration of Th17 lymphocytes in the anterior segment of OAB-subjected eyes from days 7 to 28 by immunofluorescence. Finally, we demonstrated the increased frequency of the Th17 population and elevated Th17/Treg ratio in the later phase of OAB using flow cytometry.

OAB is a detrimental event to the subjected eyes. It impacts both appearance and function, causing many fatal ocular complications [5,6,7,8]. The severe damage to the ocular surface is undoubtedly sight-threatening, resulting in corneal melting, exhaustion of limbal stem cells and eventually the decompensation of the ocular surface [4]. Intraocular inflammation after OAB is another unignorable factor in several lethal disorders, including secondary glaucoma [1,2], complicated cataract, RGCs apoptosis [6,7,8], disfunction of the ciliary body, and even bulbi phthisis [39]. Thus, controlling intraocular inflammation is essential to restoring visual function in OAB patients.

In contrast to mechanical, acidic, or thermal agents, inflammation caused by alkali stimuli can present prolonged or recurrent characteristics after the acute phase, despite the proper administration of systemic and topical corticosteroids [16]. We observed inflammatory manifestations in the anterior segment in both the acute and later phases of our rat OAB model. Histological examination revealed inflammatory cell infiltration and fibrin exudation, which confirmed prolonged anterior intraocular inflammation. It was reported that the expression of IL-1β, IL-6, and IL-10 in rat corneas increased immediately after alkaline exposure, peaked within 1 day and returned to normal in the first 2 days [11]. The level of IL-1β in corneas increased again on the 5th day, and TNF-α started rising late on the 14th day [11]. We found similar trends in the intraocular expression of IL-1β, IL-6, and IL-10 in the early phase in our experiment, but the second elevation of IL-1β could not be detected. The dynamic changes in intraocular TNF-α showed a pattern similar to that of IL-6 and IL-10 in our study, without delayed elevation. This is consistent with previous reports. These differences might be due to the imparity in cytokine expression among different tissues from which intraocular fluid and ciliary bodies were collected, rather than the corneas. Based on the persistently low levels of intraocular IL-1β, IL-6, and TNF-α in the later phase, we hypothesized that other factors might contribute to prolonged intraocular inflammation. The increased expression of IL-17A indicates the possibility of Th17 lymphocyte involvement.

Adaptive immunity and T lymphocytes have been shown to contribute to the pathological processes of corneal injury and intraocular inflammation after OAB [16,18]. Moreover, Zhao et al. determined that CD4^+^ T lymphocytes exceeded CD8^+^ T lymphocytes in number over time, and this phenomenon was more obvious in the iris than in the cornea [18]. CD4^+^ T cells were still detected in the anterior chamber in the later period when inflammatory destruction of cornea gradually diminished. This implied that prolonged anterior intraocular inflammation mediated by CD4^+^ T lymphocytes, rather than corneal dysfunction, was the continuous pathologic factor in subsequent multiple intraocular disorders. Herein, we demonstrated, using immunofluorescent staining, that some of the infiltrated CD4^+^ T lymphocytes in the anterior segment of OAB-subjected eyes could be Th17 cells. CD4 and IL-17A double positive cells were found in the anterior chamber from day 7 to day 28 after modeling. This is consistent with the observation of CD4^+^ T lymphocyte infiltration in Zhao’s research [18]. Th17 activation and Th17/Treg imbalance were demonstrated by flow cytometry, as the frequency of Th17 population and Th17/Treg ratio increased in the submaxillary and cervical lymph nodes and in the spleen. At the end of our observation, the frequency of the Th17 population returned to near normal, with a descending trend of intraocular transcriptional level of IL-17. Infiltration of Th17 cells in the anterior chamber and elevated protein level of IL-17 were still detected at the 28th day. This implied that the intraocular inflammation would cease later than the downregulation of Th17 in lymph nodes and in the spleen. Thus, Th17 activation and Th17/Treg imbalance could be candidates for therapeutic targets to alleviate prolonged anterior intraocular inflammation.

Th17 activation and Th17/Treg imbalance are closely related to the aggravation of experimental autoimmune uveitis (EAU) [36]. In EAU model, the frequency of Th17 and Treg populations together with Th17/Treg ratio began to increase on the 6th day after induction and peaked on the 12th day in eye tissue, lymph nodes, and spleen [36]. In the rat OAB model, we found a double rising trend of the Th17 population and Th17/Treg ratio with an unchanged Treg frequency in both lymph nodes and spleen. The first peak was on the 3rd day and the second rise occurred on the 14th and 21st days. Variations in Th17 frequency and Th17/Treg ratio were smaller than those in EAU. The differences between OAB and EAU models is likely related to the different patterns of their pathophysiological processes. In the EAU model, intradermal injection of purified retinal antigens induced a prominent systemic immune response and acute severe pan-uveitis, specifically targeting the retina and uvea [40]. As a strong local stimulus, alkaline chemicals would alternatively lead to the extensive destruction of various ocular tissues and an intense non-specific immune reaction against necrotic fragments or denatured proteins. Antigen presentation and Th17/Treg regulation may be more complex in the OAB model. In addition, the increased frequency of the Th17 population and Th17/Treg ratio in the spleen indicated probable systemic immune activation. This phenomenon might be associated with vascular injury and the breakdown of the blood–aqueous barrier, so that abundant necrotic fragments or denatured proteins could be released into circulation soon after alkaline exposure.

Innate and adaptive immunity together build a complex defense network against exogenous vulnerates, involving numerous immune cells, cytokines, and chemokines. Neutrophils and macrophages infiltrate the anterior segment several hours after OAB as dominant immune cells in the acute phase [11,18]. Macrophages continue to play a role in anterior inflammation within the first two weeks and contribute to retinal damage [7]. CD4^+^ and CD8^+^ T lymphocyte infiltration indicated the participation of adaptive immunity, while CD4^+^ T helper cells prevailed in the chronic stage [18]. Our results show the involvement of Th17 lymphocytes in prolonged anterior intraocular inflammation after OAB. However, Th1/Th2 responses might still play an important role. Further studies are needed to determine the proportion of each lineage of T cells, and macrophages in different stages of OAB, and to elucidate whether and when Th17 lymphocytes dominate the pro-inflammatory function.

## 4. Methods and Materials

### 4.1. Animals and OAB Model

Adult Sprague Dawley (SD) rats (6–8 weeks, 180–220 g; half male and half female, Laboratory of Zhongshan Ophthalmic Center, Guangzhou, China) were randomly divided into the following two groups: a normal control group and an OAB group. All animal experiments were performed in accordance with the guidelines of the Association for Research in Vision and Ophthalmology (ARVO) on the use of animals in research, and approved by the Zhongshan Ophthalmic Center Animal Care and Use Committee, Sun Yat-sen University, Guangzhou, China. For OAB modeling, rats were anesthetized using an intraperitoneal injection of 1.5% pentobarbital sodium (40 mg/kg, Sigma-Aldrich, St. Louis, MO, USA). The right eye was selected for the experiment. A drop of topical proparacaine was applied to the ocular surface. A 4.0-mm-diameter round filter paper was submerged in 1M NaOH for 10 s and placed on the central cornea for 40 s. After removing the alkaline stimulus, each experimental eye was promptly washed with 60 mL sterile saline. Normal control eyes were washed with 60 mL sterile saline only, without receiving any alkaline impact.

### 4.2. Slit-Lamp Examination

The anterior segment of the subject eye of each rat was evaluated and recorded by slit-lamp biomicroscopy (SL-D7/DC-3/IMAGEnet, Topcon) before and 1, 3, 7, 14, 21, and 28 days after modeling (*n* = 6 at each time point). After anesthesia, the rats were fixed in a suitable position for observation and photography.

### 4.3. Hematoxylin and Eosin Staining and Immunofluorescence

The rats were ethically euthanized at selected time points. For histological observation, enucleated eyes (*n* = 6 at each time point) were fixed in 10% formaldehyde, dehydrated with gradient alcohol, and embedded in paraffin. Sections of 5 μm were made and deparaffinized before treatment with hematoxylin and eosin (H and E). For immunofluorescence, the eyeballs were fixed in 4% paraformaldehyde (PFA) after enucleation overnight, dehydrated in 30% sucrose, and embedded in OCT compound. Serial 5 μm sections were fixed with 4% PFA for 15 min and permeabilized with 0.1% Triton-X-100 for 15 min at room temperature (RT). Sections were blocked with 5% bovine serum albumin and incubated with primary antibodies against CD4 (1:50, NBP1-19371, Novus Biologicals, Centennial, CO, USA) and IL-17A (1:50, sc-374218, Santa Cruz, CA, USA) at 4 °C overnight. Alexa Fluor 488 donkey anti-rabbit IgG (1:1000, ab150073, Abcam, Cambridge, UK) and Alexa Fluor 647 goat anti-mouse IgG H&L (1:1000, ab150115, Abcam, Cambridge, UK) were used as secondary antibodies at 37 °C for 1 h. Nuclei were stained with 4,6-diamino-2-phenylindole (DAPI) for 10 min at RT. The specimens were washed in phosphate-buffered saline, mounted onto coverslips, and analyzed using confocal microscopy (Carl Zeiss, Oberkochen, Germany).

### 4.4. Quantitative Real-Time PCR

Total RNA of ciliary bodies at different timepoints (*n* = 6 at each timepoint) was extracted using TRIzol reagent (Takara, Kustatsu, Japan) and reverse-transcribed to cDNA using PrimeScript™RT reagent kit (Takara, Japan). PCR primers were designed based on the NCBI GenBank database (Table 1). Gene expression levels were measured using a LightCycler 480 II system (Roche, Basel, Switzerland). The PCR amplification protocol consisted of 95 °C for 5 min, followed by 40 cycles of 95 °C for 5 s and 60 °C for 15 s. GAPDH was used as a reference gene, and target gene expression was calculated using 2^−ΔΔCq^ method.

### 4.5. Cell Processing and Flow Cytometry

The spleen (SP) and ipsilateral submaxillary and cervical lymph nodes (LN) from both OAB and normal control rats were separated at each selected timepoint (*n* = 6). Each tissue sample was ground to isolate cells and filtered using a cell strainer to obtain single-cell suspensions. Cells were plated in a 96-well plate (approximately 5 × 10^5^ cells/well) and stimulated in a culture medium containing phorbol myristate acetate (PMA, 50 ng/mL, Sigma-Aldrich), ionomycin (1 μg/mL, Sigma-Aldrich, St. Louis, MO, USA), and brefeldin A (BFA, 5 μg/mL, Sigma-Aldrich, St. Louis, MO, USA) in a 10% CO_2_ incubator at 37 °C for 4h for intracellular cytokine staining. LIVE/DEAD (1:1000, Thermo Fisher Scientific, Waltham, MA, USA) was used to distinguish living cells. Subsequently, the surface markers anti-rat CD3-PerCP-eFluor^®^ 710 (1:100, 46-0030-80, eBioscience, San Diego, CA, USA), CD4-FITC (1:100, 11-0040-82, eBioscience, San Diego, CA, USA), CD25-PE (1:100, 12-0390-82, eBioscience, San Diego, CA, USA), and CD45-eFluor^®^ 450 (1:100, 48-0461-80, eBioscience, San Diego, CA, USA) were used to label all the cultured cells. Then, the cells were washed, fixed, permeabilized with buffer (TNB-0607-KIT, Tonbo Biosciences, San Diego, CA, USA), and intracellularly stained with phycoerythrin (PE)-conjugated antibodies against IL-17A (1:80, 25-7177-80, eBioscience, San Diego, CA, USA) and APC-conjugated antibodies against Foxp3 (1:80, 17-5773-80, eBioscience, San Diego, CA, USA). The labeled cells were analyzed using a flow cytometer (FACSCalibur, BD Bioscience, San Jose, CA, USA) and acquisition and analysis software (CellQuest, Becton Dickinson, Franklin Lakes, NJ, USA). All data were obtained and analyzed using the FlowJo software (Tree Star, Ashland, OR, USA).

### 4.6. Determination of Cytokine Levels in Intraocular Fluid

Intraocular fluid was collected from rats at each time point (*n* = 6). Levels of IL-1β, IL-6, TNF-α, IL-10, and IL-17A were quantified using a rat cytokine/chemokine magnetic bead panel kit (Millipore, Billerica, MA, USA) according to the manufacturer’s instructions. All samples were centrifuged to remove debris and diluted at a ratio of 1:2 according to the preliminary experiments. Then, 25 μL aliquots of diluted samples, controls, or standards were added to specific wells and incubated for 2 h at RT. Subsequently, the plate was washed twice and incubated with 25 μL of detection antibodies in each well at RT for 1 h, followed by streptavidin-phycoerythrin for 30 min. After the final wash, 125 μL of sheath fluid was added to each well and the plate was evaluated using a Luminex 200™ instrument. Data were analyzed using the Milliplex Analyst software (Millipore, Billerica, MA, USA). The results are presented as picograms per milligram (pg/mg).

### 4.7. Statistical Analysis

Statistical analyses were performed using the Prism software (version 8.0; GraphPad, Inc., La Jolla, CA, USA). All data are presented as mean ± standard deviation (SD) of at least three independent experiments. One-way ANOVA followed by Bonferroni’s post hoc test was used for multiple comparisons among different time points when the indices were normally distributed. The Mann–Whitney U test was used for non-parametric testing of ordinal data. *p* < 0.05 was considered statistically significant.

## 5. Conclusions

Our study showed that Th17 activation and Th17/Treg imbalance are involved in prolonged anterior intraocular inflammation after OAB. The specific pro-inflammatory effect of Th17 lymphocytes and their interaction with other immune cells in the later phase of OAB need further investigation.

## Figures and Tables

**Table 1:** PCR primers used in the study.

Gene		Primers
IL-1β	forward	GCAGCTTTCGACAGTGAGGAGA
	reverse	CAATAGAGGAAACGCAGGTG
IL-6	forward	CAGCGATGATGCACTGTCAGA
	reverse	GGAGAGCATTGGAAGTTGGG
TNF-α	forward	GCCACCACGCTCTTCTGTCTA
	reverse	CGCTTGGTGGTTTGCTACGA
IL-10	forward	TGCTCTTACTGGCTGGAGTG
	reverse	CCTGGGGCATCACTTCTACC
IL-17	forward	ATCCAGCAAGAGATCCTGGT
	reverse	CAATAGAGGAAACGCAGGTG
GAPDH	forward	GGATGGAATTGTGAGGGAGA
	reverse	GTGGACCTCATGGCCTACAT

## Data Availability

Data is contained within the article or Supplementary Materials.

## Supplementary Materials

The following supporting information can be downloaded at: https://www.mdpi.com/article/10.3390/ijms23137075/s1.
