# Supplementary material for: Th17 Activation and Th17/Treg Imbalance in Prolonged Anterior Intraocular Inflammation after Ocular Alkali Burn
Source: Int J Mol Sci. 2022 Jun 25;23(13):7075. doi: 10.3390/ijms23137075 (PMC9266712; doi:10.3390/ijms23137075)
Supplement: Supplementary file 1 [file ijms-23-07075-s001.zip › Supplementary Figure S1.pdf]

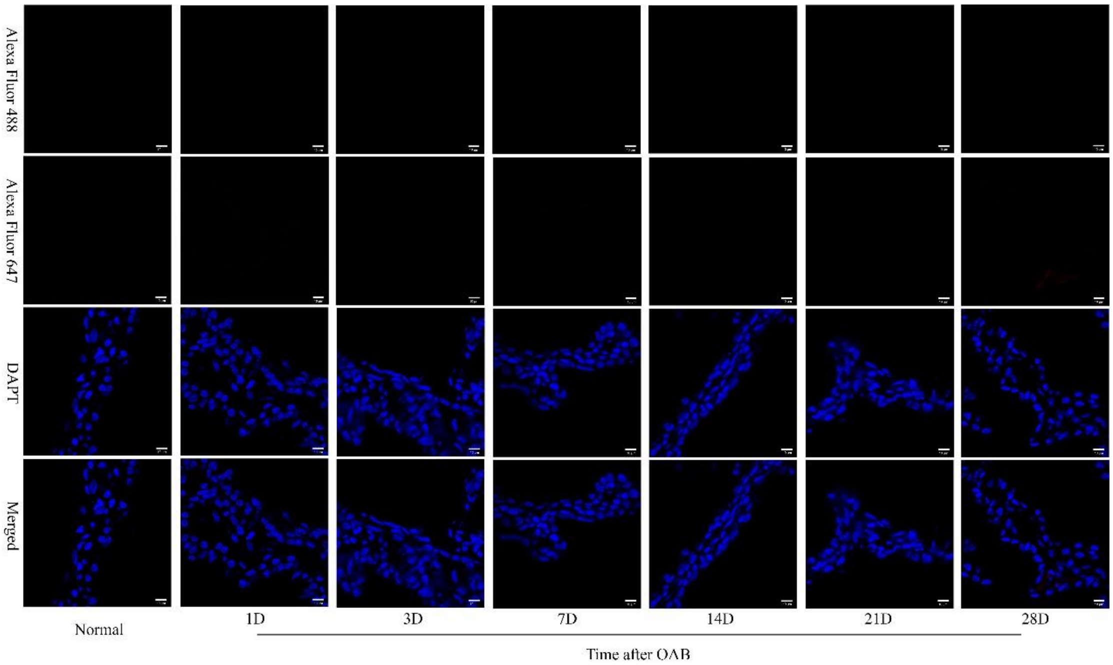

Supplement Figure S1. Immunofluorescence negative control of CD4 and IL-17A. Negative control of CD4 (Alexa Fluor 488) and IL-17A (Alexa Fluor 647) in the normal control and OAB-subjected eyes on the 1st, 3rd, 7th, 14th, 21st, and 28th day post modeling (63X, n=6 for each time point). All the sections were incubated with secondary antibodies as mentioned in the manuscript at 37 °C for 1h after treated with PBS at 4 °C overnight. Primary antibody against CD4 and IL-17A were not used. Cell nuclei were stained with DAPI (blue). Scale bar: 10 μm.
